# Abnormal Activation and Connection in Middle Frontal Gyrus: A Potential Imaging Feature for Facial Synkinesis Comorbid Depression

**DOI:** 10.1155/da/1705507

**Published:** 2026-04-25

**Authors:** Haonan Guan, Luyao Wang, Fan Dong, Wenjing Hu, Zhilin Zhang, Jinglong Wu, Yue Lu, Wei Ding, Jiehui Jiang

**Affiliations:** ^1^ Department of Plastic and Reconstructive Surgery, Shanghai Ninth People’s Hospital, Shanghai Jiaotong University School of Medicine, Shanghai, 200011, China, shsmu.edu.cn; ^2^ Institute of Biomedical Engineering, School of Life Sciences, Shanghai University, Shanghai, 200444, China, shu.edu.cn; ^3^ Research Center for Medical Artificial Intelligence, Shenzhen Institute of Advanced Technology, Chinese Academy of Sciences, Shenzhen, 518055, Guangdong, China, cas.cn; ^4^ School of Medical Technology, Beijing Institute of Technology, Beijing, 100081, China, bit.edu.cn; ^5^ Department of Neurology, Shanghai Changhai Hospital, Shanghai, China, chhospital.com.cn

**Keywords:** brain activation, depression, functional connectivity, task fMRI

## Abstract

Patients with facial synkinesis (FS) comorbid depression may experience aggravated symptoms of perceived synkinesis and poorer recovery outcomes. Exploring imaging features associated with depressive symptoms could help us better understand disease conditions and formulate appropriate rehabilitation plans. Although abnormal activation in the middle frontal gyrus (MFG) has been reported in depression patients, whether it represents an imaging correlate of depressive symptoms in patients with FS is still unknown. Therefore, a total of 52 participants (20 normal controls [NCs], 32 patients) with both task and resting‐state functional magnetic resonance imaging (rs‐fMRI) data were included in this study. Comorbid depression was assessed with the Diagnostic and Statistical Manual of Mental Disorders, 5th edition (DSM‐V), and depressive symptom severity was measured with the Beck Depression Inventory‐II (BDI‐II). We investigated brain activation during motor tasks (smiling, blinking, and grinning). In addition, the seed‐based functional connectivity (FC) between the MFG and other brain areas was calculated in the resting state. Additionally, we analyzed the associations between brain activity and clinical scale scores. The results revealed reduced activation in the sensorimotor and MFG during affected side movements across all tasks (voxel‐level *p* < 0.05, cluster‐level *p* < 0.05, Gaussian random field [GRF]‐corrected). Moreover, seed‐based FC between the MFG and emotion‐related areas was increased (voxel‐level *p* < 0.05, cluster‐level *p* < 0.05, GRF‐corrected), indicating impaired emotional networks in patients. Scores on the BDI‐II were negatively associated with MFG activation during tasks (R = −0.318, *p* = 0.027) and positively associated with the FC of the MFG (*R* = 0.387, *p* = 0.029). Thus, the MFG activation and connectivity may represent an increased risk for elevated depressive symptoms in patients with FS, warranting further validation in future studies.

**Trial Registration:** Chinese Clinical Trial Registry: ChiCTR1800014630.

## 1. Introduction

Facial synkinesis (FS) is a common consequence of peripheral facial nerve palsy and occurs in 55%–78% of cases [1]. Owing to impaired proprioceptive and visual feedback on the face, patients show uncoordinated activation of oral elevators and depressors. This condition results in impaired facial expressions, such as smiles, blinks, and grins, which are essential for expressing happiness or sadness [2]. Abnormal facial expressions prevent patients from expressing their true feelings and make them vulnerable to misunderstandings by others, thus being associated with an increased risk of depressive symptoms [3]. Research indicates an increased incidence of depression associated with FS, especially within 2 years after disease development [4]. Depression can influence perceived synkinesis severity and affect scores on patient‐reported outcome measures [1]. It also affects a patient’s experience in receiving treatment [5]. Exploring imaging features associated with depressive symptoms in patients with FS could facilitate a better understanding of the disease condition and the formulation of appropriate rehabilitation plans.

Currently, clinical scales are primarily used to assess depressive symptoms in patients with FS, which are more dependent on the subjective perceptions of patients. Functional magnetic resonance imaging (fMRI) data could provide quantitative indicators of brain function in both the task and the resting state. On this basis, many studies suggest that the frontal cortex is involved in emotion regulatory processes, especially its ability to modulate the activity of subcortical regions, such as the cingulate cortex [6–9]. Within the frontal cortex, the middle frontal gyrus (MFG) overlaps with the dorsolateral prefrontal cortex (DLPFC), which is often used as noninvasive neuromodulation for depression [10, 11]. In addition, several studies have shown that the MFG may play either a positive or a negative role in emotion regulation in depression. For example, Colich et al. [12] reported increased MFG activation during emotional interference, whereas Zhang et al. [13, 14] reported reduced MFG activation and increased MFG connectivity during negative emotion processing. Although fMRI studies revealed abnormal MFG activation in depression patients, prior studies on FS have focused mainly on maladaptive reorganization within motor networks. For example, Wang et al. [15] used task‐based fMRI and reported reduced activation in the cortico‐facial motor area and increased activation in the contralateral supplementary motor area (SMA), suggesting cortical reorganization. Zhang et al. [16] reported that structural–functional coupling is markedly reduced, whereas functional connectivity (FC) shows compensatory and significant enhancement in FS patients. Guntinas‑Lichius et al. [17] also reported that patients with FS exhibit abnormal activations and increased FC. In summary, previous studies have shown that affective network dysfunction occurs in patients with depression and that motor network abnormalities occur in patients with FS. However, the interaction between emotion‐related brain regions and FS pathology, which may underlie the high prevalence of comorbid depression, remains largely unexplored.

Therefore, our study focused on brain activity during smile, blink, and grin tasks in FS patients. These tasks reflect the neural control of facial expressions. Healthy individuals can perform these expressions normally, whereas patients with FS show abnormal or involuntary movements. Specifically, the smile task involves the buccal branch, the blink task involves the zygomatic branch, and the grin task involves the marginal mandibular branch. Each of these branches plays a different role in emotional expression. In addition, we performed seed‐based analysis by resting‐state fMRI (rs‐fMRI) to investigate the FC between the MFG and the whole brain. Furthermore, we examined the relationships between brain activation and the use of clinical scales. Studies have shown that there is a bidirectional interaction between the sensorimotor system and brain emotional and higher cognition networks [18, 19]. We hypothesize that patients with FS comorbid depression exhibit abnormal alterations in both the activation and FC of the MFG, characterized by atypical increases or decreases in task‐evoked activation and elevated resting‐state connectivity. We further predict that these changes will correlate with higher depression scores and greater severity of FS. Our findings aim to provide multimodal evidence that MFG dysfunction may represent a candidate imaging feature associated with depressive symptoms in patients with FS and offer valuable insight for developing potential intervention targets.

## 2. Materials and Methods

### 2.1. Participants

A total of 52 participants were initially recruited, including 20 normal controls (NCs) and 32 patients. Among the patients with FS (15 with right synkinesis and 17 with left synkinesis), participants were further stratified into a high depressive symptom group (FS‐HDS, *n* = 14) and a low depressive symptom group (FS‐LDS, *n* = 18). The grouping was based on assessment with the Diagnostic and Statistical Manual of Mental Disorders, 5th edition (DSM‐V) criteria for depression and the Beck Depression Inventory‐II (BDI‐II) scale for symptom severity. Participants meeting DSM‐V criteria for depression were further classified using a BDI‐II scale, with a cut‐off score of 13 following established criteria in previous studies [20]. After quality control procedures, three participants were excluded due to excessive head motion or incomplete imaging data. The final sample included 19 NC participants, 14 FS‐HDS participants, and 16 FS‐LDS participants. All participants were recruited from the Ninth People’s Hospital, School of Medicine, Shanghai Jiao Tong University, China. The demographic information of all the participants is summarized in Table 1. This study received approval from the Ethics Committee of Shanghai Jiao Tong University (Approval No. 2017‐365‐T267). Written informed consent was obtained from all volunteers and patients prior to participation.

**Table 1:** Demographic data of the NC and FS groups.

Demographic and clinical characteristics	NC	FS			*p*‐Value
		All	FS‐HDS	FS‐LDS	
Gender (M:F)	3:16	11:19	4:9	7:10	0.115^†^
Age (year)	26.15 ± 3.75	33.43 ± 8.64	31.61 ± 7.68	34.82 ± 9.28	~ 0.001^‡^
Course of disease (year)	—	6.100 ± 7.45	5.75 ± 7.60	10.39 ± 10.73	—
Sunnybrook	—	42.10 ± 14.08	40.50 ± 13.59	38.75 ± 17.34	—
Synkinesis	—	8.06 ± 2.18	8.07 ± 2.48	8.18 ± 1.29	—
Resting symmetry	—	5.83 ± 4.74	6.07 ± 5.70	7.18 ± 5.53	—
Symmetry of voluntary movement	—	56.00 ± 11.88	54.64 ± 13.56	54.00 ± 19.13	—
Beck Depression Inventory‐II	—	17.04 ± 12.22	6.20 ± 4.70	26.90 ± 7.38	—

*Note:* The measurement data are presented as the means ± standard deviations. Statistical analysis between the NC and FS groups was performed with the following tests: chi‐square test (†) and the Mann‒Whitney *U* test (‡).

The participants in the NC group were healthy volunteers without any neurological or mental disorders. The inclusion criteria for patients were as follows: (1) a history of oral‐ocular synkinesis for more than 9 months, (2) absence of nerve transposition, and (3) ability to follow instructions. The exclusion criteria for both groups were as follows: (1) confirmed or suspected history of cardiopulmonary failure, (2) other psychiatric disorders, (3) concurrent peripheral neuropathy, and (4) contraindications to MRI investigation [15]. We collected clinical scales for each patient, including the Sunnybrook Facial Grading System and the BDI‐II. The Sunnybrook facial grading system includes a total score (Sunnybrook) and three domains: the synkinesis domain, the resting symmetry domain, and the voluntary movement domain.

### 2.2. Experimental Design

Each participant underwent one rs‐fMRI scan and six motor task fMRI scans. The motor tasks included (1) blinking the right eye, (2) blinking the left eye, (3) smiling the right half of the face, (4) smiling the left half of the face, (5) grinning the right half of the face, and (6) grinning the left half of the face. Unilateral eye blinking involved rhythmic blinking of one eye. In the smiling task, participants were instructed to smile with unilateral muscle contraction innervated by the buccal branch. In the grinning task, participants were asked to stretch one side of their mouth downwards.

During motor tasks, participants rhythmically performed repetitive movements at 1 Hz guided by visual signals. The signals were projected onto a screen via a video projector and reflected onto a mirror attached to the head coil. Each action lasted 30 s and alternated with a 30‐second rest period. Each fMRI scan run comprised three blocks of motor tasks and three blocks of rest. Prior to the experiment, all the subjects received ~20 min of task training, and the facial movements of each patient were recorded by a camera.

### 2.3. Data Acquisition and Preprocessing

All imaging data were obtained with a 3.0T MRI scanner (GE Discovery MR 750) and 8‐channel phased array coils. None of the participants were taking medications known to affect mood or central nervous system function (e.g., antidepressants or anticholinergic agents) at the time of scanning. T1‐weighted MRI images were acquired via a magnetization‐prepared rapid gradient echo sequence with the following parameters: repetition time (TR) = 8.156 ms, echo time (TE) = 3.18 ms, inversion time = 450 ms, matrix size = 256 × 256, field of view (FOV) = 256 × 256 mm^2^, flip angle (FA) = 12°, and voxel size = 1 × 1 × 1 mm^3^. The task fMRI data were acquired via a gradient‐echo echo‐planar imaging (EPI) sequence with the following parameters: TR = 3000 ms, TE = 30 ms, slice thickness = 3.2 mm, slice number = 43, matrix size = 64 × 64, FOV = 240 × 240 mm^2^, FA = 77°, voxel size = 3.4375 × 3.4375 × 3.2 mm^3^, and time points = 60. The rs‐fMRI data were acquired via an EPI sequence with the following parameters: TR = 2000 ms, TE = 30 ms, slice thickness = 3.2 mm, slice number = 43, matrix size = 64 × 64, FOV = 240 × 240 mm^2^, FA = 90°, voxel size = 3.4375 × 3.4375 × 3.2 mm^3^, and time points = 240.

Preprocessing was performed via Statistical Parametric Mapping 12 (SPM12, the Wellcome Department of Neurology, London, UK) software implemented in MATLAB 2018b (Mathworks Inc.), including slice timing correction, spatial realignment, coregistration, normalization to Montreal Neurological Institute (MNI) space on the basis of the average blood oxygenation level dependent (BOLD) image resliced in realignment, and smoothing with a 6 mm full‐width at half‐maximum (FWHM) isotropic Gaussian kernel. We aligned all functional volumes of each participant to the first image of the series using rigid‐body transformations. This process estimates translations and rotations to minimize differences between volumes, producing motion‐corrected images and six motion parameters for later quality assessment and artifact control. Motion‐related artifacts were controlled by setting thresholds for head translations (≤2 mm) and rotations (≤2°), excluding runs exceeding these thresholds, and including head motion parameters as regressors in the general linear model (GLM) to reduce confounding effects. For rs‐fMRI, linear detrending and bandpass filtering (0.01–0.1 Hz) were also performed. We excluded three participants whose head motion exceeded 2 mm displacement or 2° rotation. The following analysis included only 19 NC and 30 patients.

During the task‐based fMRI analysis, task compliance was evaluated through reaction time, accuracy, and real‐time observation by the experimenters, and all included participants met the compliance criteria. Comparisons of task performance between FS and NC groups showed no significant differences, indicating that the task difficulty was appropriate for all participants. The details of head motion are reported in the Table S2 and S3). Specifically, some participants’ data were further excluded from specific motor tasks due to excessive head motion: one participant was excluded from each of the left blink motor, left grin motor, and right blink motor tasks, and two participants were excluded from the right grin motor task. Detailed information on the final participant numbers included in each task analysis for all groups can be found in Table S4.

### 2.4. Task Activation Analysis

First‐level analysis uses a GLM to estimate stimulus‐induced activation [21]. The BOLD response was modeled as neural activity convolved with a canonical hemodynamic response function. Six head motion parameters were included as nuisance regressors. For each participant, six task conditions were modeled, corresponding to three facial expression tasks (smile, blink, and grin) performed on the left and right sides of the face. Each condition was treated as an independent regressor in the first‐level GLM and convolved with the canonical HRF. The baseline was defined as rest periods between task blocks when participants were instructed not to perform any facial movements. Contrast images were generated for each task versus baseline (e.g., smile > baseline, blink > baseline, grin > baseline).

The contrast images (.con) from the first‐level analysis of the right FS patient group were flipped for consistency and used for second‐level group statistics. After that, the affected side corresponds to left face movements, and the unaffected side corresponds to right face movements. For each task, we performed second‐level analyses to identify brain regions showing significant group differences. Two‐sample *t* tests were used to compare the NC and FS groups, as well as the FS‐HDS and FS‐LDS groups. Age and sex were included as covariates in the model to control for their potential effects. To correct for task‐related multiple comparisons at the voxel level, we applied a Gaussian random field (GRF) correction at voxel‐level *p* < 0.05 and cluster‐level *p* < 0.05. To explore the synkinesis‐related characteristics, activation similarity within the right MFG was calculated as the Spearman correlation coefficient between voxel‐wise activation values of different tasks within this ROI on the affected face side, reflecting the spatial pattern consistency of activation across tasks.

### 2.5. Seed‐Based FC Analysis

For the rs‐fMRI analysis, the seed region in the MFG.R was defined as the area showing significant differences in activation between the NC group and patient group on the affected side across the three tasks, with the center located at the MNI coordinates (32, 44, 29). This seed region comprised a total of 142 gray matter voxels. FC was calculated via Pearson correlation between the time series of the seed and whole‐brain voxels, allowing assessment of whether the MFG.R exhibits coordinated spontaneous signals with other brain regions. Fisher’s Z transformation was used for normalization. We first obtained voxel‐wise FC maps and applied GRF correction to identify significant FC clusters. To determine the GM ROIs with significant differences, the significant clusters were parcellated via the AAL atlas [22, 23]. In addition to the NC‐FS comparison, the same pipeline was used to generate ROI‐level FC differences for the FS‐HDS and FS‐LDS groups to identify abnormal connections.

### 2.6. Statistical Analysis

MATLAB and GraphPad Prism version 9.0.0 for Windows were used for the statistical analyses. The Anderson‒Darling test was used to assess the normality of the data distribution. Chi‐square tests, two‐sample *t* tests, and Mann‒Whitney *U* tests were used to explore differences in clinical characteristics between the NC and patient groups. To control for the effects of age and sex, we included them as covariates in both the activation and FC analyses. For task activation differences, we displayed significant areas (>50 voxels) corrected for GRF correction (voxel‐level *p* < 0.05, cluster‐level *p* < 0.05). In the visualization of task‐based results, we presented positive t values to indicate greater activation in the NC group than in the FS group and in the FS‐LDS group than in the FS‐HDS group. For FC differences, we used GRF correction (voxel‐level *p* < 0.05, cluster‐level *p* < 0.05) to explore abnormal connections. In the visualization of the resting‐state FC results, positive t values were used to represent stronger connectivity in FS patients than in NC and in FS‐HDS patients than in FS‐LDS patients.

In addition, we explored the relationships between clinical scales and brain activation intensity via Spearman correlation. The clinical scales included the Sunnybrook Facial Grading System and the BDI‐II. Similar analyses were performed between the clinical scales and FC. The correlation analyses were adjusted for age and sex, with *p* < 0.05 after FDR correction. Before the correlations were computed, the activation intensity and FC values were standardized via *z* scores across tasks and participants to reduce variance and ensure comparability.

## 3. Results

### 3.1. Demographic Data

The demographic information of all participants is summarized in Table 1. All patients presented with FS, with synkinesis scores ranging from 3 to 13 (mean ± SD: 8.067 ± 2.180). The average BDI‐II score of the FS‐HDS group was 26.909 ± 7.381, which was above the clinically significant cut‐off of 13. There was a significant difference in age between the NC and patient groups (*p* < 0.001), but there was no difference in sex distribution. To control for potential confounding effects of age and sex on imaging measures, these variables were included as covariates in all difference analyses.

### 3.2. Differences of the Activation Intensity

**Figure 1:**
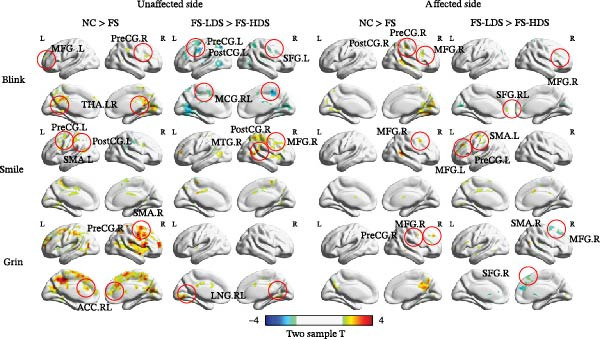
Differences in activation between the NC and FS groups or between the FS‐HDS and FS‐LDS groups. To ensure the uniformity of the results, the images of the right FS patient group were flipped. After that, the affected side corresponds to left facial movements, and the unaffected side corresponds to right facial movements. THA, thalamus; TPS, temporal pole superior; ITG, inferior temporal gyrus; MFG, middle frontal gyrus; MTG, middle temporal gyrus; PostCG, postcentral gyrus; PreCG, precentral gyrus; MCG, median cingulate and paracingulate gyrus; SMA, supplementary motor area.

**Figure 2:**
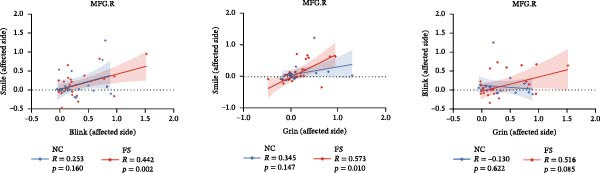
Similarity of activation intensity across the three tasks on the affected face side. Spearman correlation (*r*) was calculated between the activation intensity of the control and patient groups.

The activation maps for all six motor tasks across both groups are shown in Figure 1 and Table 2. Two‐sample *t* test results between the NC and FS groups revealed differences in activation, mainly in the pre/postcentral gyrus (PreCG/PostCG), indicating FS‐related motor and somatosensory abnormalities, as well as in several emotion‐related areas, including the thalamus (THA) and MFG. For the FS‐LDS vs. FS‐HDS comparison, the analyses focused on depression‐related differences, with altered activation patterns primarily involving the MFG and SFG. Moreover, Figure 2 further presents a comparison of the activation intensity on the affected face side among the three tasks. In the patient group, the activation intensity on the affected face side was significantly positively correlated with smiling and blinking tasks (*p* = 0.002) and between smiling and grinning tasks (*p* = 0.010). The correlation between blinking and grinning tasks was not significant (*p* = 0.085).

**Table 2:** Clusters showed inter‐group differences.

Contrast	Task	Region	*x*	*y*	*z*	Voxel	Peak intensity	Corrected cluster *p*	Corrected voxel *p* (mean)
NC vs. FS	Right blink	Frontal_Mid_L/Frontal_Sup_L	−18	57	3	191/244	3.75	0.01	0.04
		Thalamus	6	−42	15	306	4.46	0.02	0.04
		Precentral_L	−33	−6	42	100	3.17	0.01	0.03
		Frontal_Mid_R/Precentral_R	48	10	43	84/120	3.38	0.02	0.05
	Right smile	Precentral_L	−15	6	42	97	3.82	0.03	0.03
		Postcentral_L	−9	−36	18	101	3.35	0.04	0.06
	Left smile	Frontal_Mid_R/Precentral_R	45	12	45	177/68	3.40	0.04	0.05
		Frontal_Sup_R	15	57	30	102	2.92	0.03	0.06
	Right grin	Precentral_RL/Supp_Motor_Area_RL	−18	−36	45	306/239	4.80	~ 0.01	0.02
		ACC_RL/Frontal_Sup_R	24	30	9	71/141	4.49	~ 0.01	0.02
	Left grin	Frontal_Mid_R/Frontal_Sup_R/Precentral_R	42	−6	36	271/171/125	3.67	0.03	0.06
FS‐LDS vs. FS‐HDS	Right blink	Frontal_Mid_R	42	45	15	63	−2.96	0.03	0.05
		Precentral_L/Postcentral_L	−52	−7	40	222/124	−3.11	0.02	0.05
	Left blink	Frontal_Sup_RL	−6	60	−3	31	2.57	0.04	0.07
		Frontal_Mid_L	42	28	22	19	2.20	0.09	0.07
	Right smile	Postcentral_R/Frontal_Mid_R	26	27	31	336/154	3.76	0.03	0.04
		Temporal_Mid/Temporal_Sup	63	−45	15	69/64	4.08	0.02	0.05
	Left smile	Frontal_Mid_L/Precentral_L/Supp_Motor_Area_L	−42	33	36	407/136/124	3.70	0.02	0.04
	Right grin	Lingual_RL	−6	−72	−6	357	4.11	0.01	0.05
	Left grin	Frontal_Sup_R/Frontal_Mid_R	12	24	12	331/66	−3.70	0.02	0.04

*Note:* Cingulum_Mid, median cingulate and paracingulate gyrus; Fusiform_L/R, left/right fusiform gyrus; Occipital_Mid_L/R, left/right middle occipital gyrus; Occipital_Sup_R, right superior occipital gyrus; Frontal_Mid_L/R, left/right middle frontal gyrus; Precentral_L/R, left/right precentral gyrus; Frontal_Inf_Tri_L/R, left/right inferior frontal gyrus, triangular part; Postcentral_L/R, left/right postcentral gyrus; Frontal_Sup_L, left superior frontal gyrus, dorsolateral; Insula_L, left insula; Frontal_Inf_Oper_R, right inferior frontal gyrus, opercular part; ParaHippocampal_R, right parahippocampal gyrus; Supp_Motor_Area_L/R, left/right supplementary motor area.

In the blinking task, significant areas (>50 voxels) were identified via two‐sample tests (GRF‐corrected voxel‐level, *p* < 0.05; cluster‐level, *p* < 0.05). The NC and FS groups presented significant differences in the bilateral cortex, including the THA, left MFG (MFG.L), and right PreCG (PreCG.R), with increased activation in the NC group. On the affected side, the MFG.R and PreCG.R exhibited significantly decreased activation in the patient group. Notably, in the comparison between FS‐HDS and FS‐LDS groups, significant activation differences were found in the MFG, SFG, and MCG. On the affected side, the MFG.R and SFG.RL showed significantly decreased activation in the FS‐HDS group.

For the smiling task, significant areas (>50 voxels) were identified via two‐sample tests (GRF corrected voxel‐level, *p* < 0.05; cluster‐level, *p* < 0.05). The NC group exhibited more significantly increased activation in the MFG.R and PreCG/PostCG. The unaffected face side of the patient group showed an activation pattern similar to that of the NC group but with decreased activation in the left SMA. In the comparison between the FS‐HDS and FS‐LDS groups, the FS‐HDS group showed significantly decreased activation on the nonaffected side of the PostCG.R, MFG.R, and MTG.R. On the affected side, the FS‐HDS group also exhibited significantly decreased activation in the PreCG.L, SMA.L, and MFG.L.

In the grinning task, significant areas (>50 voxels) were identified via two‐sample tests (GRF‐corrected voxel‐level, *p* < 0.05; cluster‐level, *p* < 0.05). The NC group showed increased activation in the primary sensorimotor cortex and emotion‐related cortex areas such as the MFG. On the affected face side, the patient group had a significant decrease in the activation of the PreCG and MFG.R compared with the NC group. In the comparison between the FS‐HDS and FS‐LDS groups, the FS‐HDS group showed decreased activation in the nonaffected right and left lingual (LNG.RL) sides. On the affected side, the FS‐HDS group exhibited significantly increased activation in the SFG.R and MFG.R.

In the comparison between the FS and NC groups, we observed that the activation differences on the affected side showed a relatively similar spatial distribution in the MFG across different facial expression tasks (smile, blink, and grin). In contrast, when comparing the FS‐LDS and FS‐HDS subgroups, the affected‐side activation differences were mainly localized to the right MFG (MFG.R) for the blink and grin tasks, whereas for the smile task, the differences were primarily observed in the left MFG (MFG.L). These findings indicate that the spatial patterns of MFG activation differences vary depending on the specific facial task.

### 3.3. Differences of the FC Between Groups

**Figure 3:**
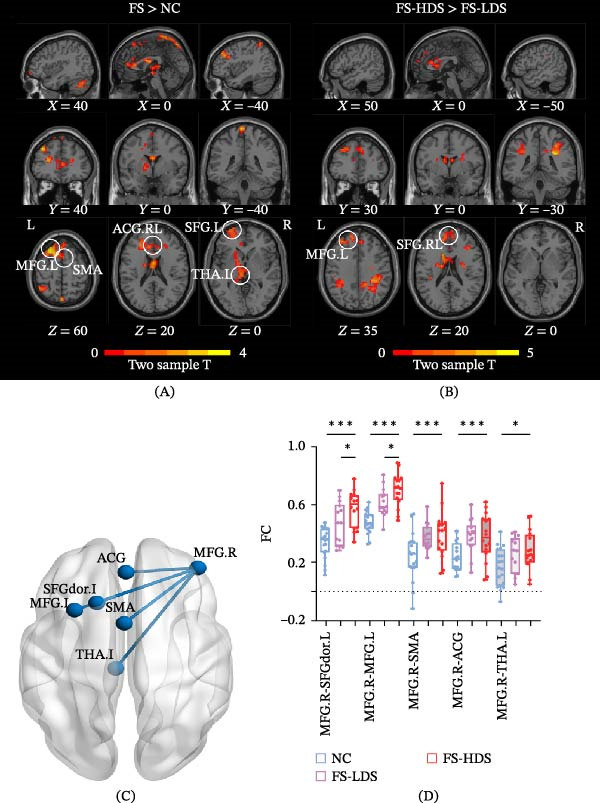
FC differences between the (A) NC and FS groups, (B) FS‐HDS and FS‐LDS groups, and (C) connectivity with significant differences. (D) Results of the seed‐based analysis of FC differences ( ^∗∗∗^ *p* < 0.001,  ^∗^ *p* < 0.05). SFGdor.L, left dorsal superior frontal gyrus; ACG, anterior cingulate gyrus; MFG.L, left middle frontal gyrus.

As all affected side results revealed significant differences in MFG activation between the NC and FS groups across all three tasks, we extracted the MFG mask on the basis of these voxels for seed‐based FC analysis. This enables us to further explore abnormal functional activation in FS patients in the resting state. The results indicated increased FC between the MFG.R and several clusters in patients (voxel *p* < 0.05, cluster‐level *p* < 0.05, GRF corrected) (Figure 3A, C), including the SMA, left MFG, left dorsal superior frontal gyrus (SFGdor.L), left THA (THA.L), and anterior cingulate gyrus (ACG). To further explore whether these FC differences were related to the depressive state in FS patients, we compared the FS‐HDS and FS‐LDS groups (Figure 3B, D). Increased FC was observed between the MFG.R and MFG.L and between the MFG.R and SFG.L (*p* = 0.049 and *p* = 0.032) in the FS‐HDS group. In contrast, no significant differences in FC were observed between the FS‐HDS and FS‐LDS groups in the SMA, ACG, or THA (*p* = 0.773, *p* = 0.868, *p* = 0.645).

### 3.4. Correlations Between MFG and Clinical Scale Scores

**Figure 4:**
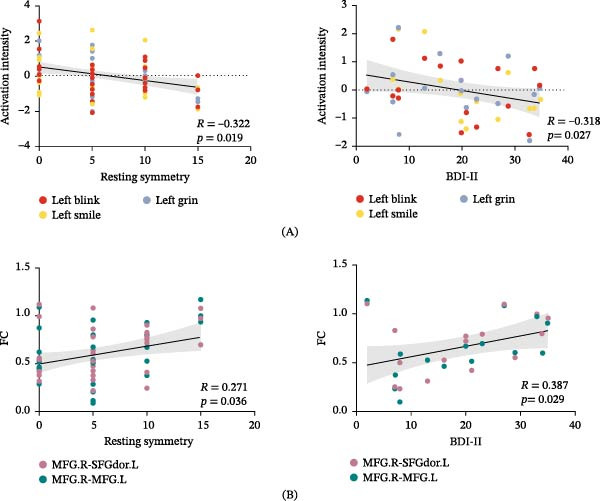
Correlation across activation intensity resting symmetry scores and BDI‐II scores. (A) Correlations between activation intensity and resting symmetry scores or between activation intensity and BDI‐II scores in the MFG. (B) Correlations between FC and resting symmetry scores or between FC and BDI‐II scores. BDI‐II, Beck Depression Inventory‐II; FC, functional connectivity.

For tasks involving the affected side, there was a significant negative correlation between the resting symmetry score and the activation intensity of the MFG.R (*R* = −0.322, *p* = 0.019) (Figure 4A). In addition, we assessed the relationship between functional activity and depression scales (BDI‐II). All movement tasks on the affected face side showed a negative correlation between BDI‐II scores and the activation intensity of the MFG.R (*R* = −0.318, *p* = 0.027) (Figure 4A). Furthermore, there was a positive correlation between the FC of the MFG.R and resting symmetry scores (*R* = 0.271, *p* = 0.036), as well as BDI‐II scores (*R* = 0.387, *p* = 0.029) (Figure 4B).

## 4. Discussion

Facial expressions serve as crucial social communication tools for conveying nonverbal emotional information. Patients with FS often experience depressive states, significantly impacting social communication and overall well‐being. This study included task fMRI to examine brain activation patterns and rs‐fMRI to assess FC. Unlike previous studies that focused mainly on motor‐related brain activation, our study uniquely investigated emotion‐related brain regions, particularly the MFG, to link FS with depressive risk. We explored whether patients with depression due to FS had impairments in MFG activation and connections in both the task and the resting state. Compared with NCs, our findings revealed reduced activation across the sensorimotor and MFG during all three motor tasks, particularly during movements of the affected side. In addition, seed‐based FC analysis demonstrated increased connectivity between the MFG and emotion‐related networks during the resting state in patients. These results enhance our understanding of neural mechanisms associated with depressive symptoms in FS and may reflect potential imaging features related to these symptoms.

Previous studies have highlighted nerve injury and regeneration at various levels of the nervous system [24–26]. Here, we focused on cortical reorganization within the central nervous system in patients with depression associated with FS. Compared with the NC group, patients exhibited reduced activation in terms of movements on the unaffected side of the face. Notably, significant differences were observed in the PreCG and PostCG areas during the blink and grin tasks, which is consistent with findings from previous meta‐analyses indicating diminished activity in the PreCG of synkinesis patients [2]. This hypoactivity in the PreCG suggests functional alterations in the motor pathway [27, 28]. Although there is evidence suggesting minimal impact on sensory afferents in FS patients [29], the reduced activation in the PostCG in our study may reflect changes in cortico‐cortical feedback pathways within the sensorimotor network [30].

In addition to the sensorimotor cortex, we detected significant differences in the activation of emotion‐related areas, including the THA, cingulate gyrus, and frontal gyrus, between the NC and FS groups. These regions are involved in emotional processing and regulation, supporting accurate emotional expression. Thalamic hypoactivity may reduce access to affective content and impair the THA–frontal gating of both salience and conscious perception [31, 32]. Dysfunction in the anterior cingulate cortex may disrupt affective appraisal and alter reward processing, both of which are important for mood regulation [33, 34]. These findings indicate that FS affects brain regions involved in emotion regulation and monitoring. Importantly, significant differences in MFG activation were detected between the FS‐HDS and FS‐LDS groups on the affected face side. This area comprises a substantial portion of the DLPFC, which often serves as a pivotal node in the central executive network crucial for emotional processing [35]. Abnormalities in the DLPFC and MFG reduce top‑down cognitive control over emotion. This may impair reappraisal and weaken the regulation of negative affect [36, 37]. Additionally, MFG activation varied across tasks, reflecting differences in cognitive or motor demands and task‐dependent variability, and future studies with larger samples are needed to confirm the stability of these patterns [38]. Furthermore, FS patients exhibited similar activation patterns in the MFG during some different facial expressions, potentially indicating deficits in expressing positive or negative emotions due to perceived inefficacy in facial movement [39]. Studies have reported increased perceived happiness and decreased sadness following depressor anguli oris excision [40, 41]. We presume that these similarities are associated with the use of facial muscles with different emotional expressions. Furthermore, the lack of correlation between the blink and grin tasks may be related to branch‐specific reinnervation [42]. These results suggest that altered MFG activation is associated with impaired emotional perception and may reflect a neural feature linked to depressive symptom severity in patients with FS.

Moreover, emotional activities engage high‐level cognitive functions such as memory, cognition, and reasoning [43], involving complex interactions across different brain regions. Our seed‐based analysis revealed significantly increased FC between MFG.R and emotional networks, such as MFG.R‐SFGdor and MFG.R‐MFG.L in the FS‐HDS group. These findings have been supported by previous studies. The MFG and SFG are fronto‐cortical nodes involved in emotion processing and top‐down regulation [44, 45]. These fronto‐cortical nodes often exhibit increased connectivity within the frontoparietal network in depression [46, 47]. Enhanced FC in rs‐fMRI may indicate compromised emotional networks across different cognitive states. Correlation analysis further suggested associations between MFG activation and FC with resting symmetry and BDI‐II scores. These results indicated that an abnormal MFG was related to higher depression scores and poorer FS. Notably, the DLPFC has been targeted in noninvasive treatment approaches for treatment‐resistant depression, with therapeutic effects linked to altered FC between the MFG and emotional network [10, 48, 49]. Our results suggest that altered FC of the MFG may be associated with depressive symptom severity in patients with FS. However, the sample size in each subgroup was small, and some results were only marginally significant. Therefore, the present results should be interpreted as exploratory, and larger, multicenter studies are required to further validate these observations.

There are several limitations in this study. Owing to the fact that task studies require patients to cooperate highly and the data are scarce, we collected relatively small sample sizes in this study, with sample sizes further reduced after subgroup analyses. Although we ensured the reliability of the data through training and strict data control, differences in age across groups could not be completely avoided. Given the relatively small sample size and the multiple task‐based and connectivity comparisons performed, potential effects of multiple comparisons cannot be fully excluded. And a part of correlation results was exploratory. Some results should therefore be interpreted with caution. Future studies with larger sample sizes are needed to further validate and extend these findings. Additionally, depression is influenced by various factors, including illness, life experiences, and the family environment. Future research could adopt a multifactorial approach to explore their correlations with synkinesis. Finally, multicenter or multispecies data are needed for comparative validation, which would increase the reliability and generalizability of our findings.

## 5. Conclusion

Investigating the neural correlates of depressive symptoms in FS patients helps reveal how impaired emotional regulation affects rehabilitation, providing conceptual insight for future research on therapeutic strategies. This study focused on exploring neural imaging features associated with depressive symptoms in patients with FS. Our findings highlight that altered MFG activation patterns during motor tasks, and FC abnormalities between the MFG and emotional networks were associated with the severity of depressive symptoms. These exploratory findings may provide theoretical insight for future research and contribute to identifying neural mechanisms underlying depressive symptoms in FS patients, potentially informing targeted intervention strategies.

## Acknowledgments

The authors would like to thank all the participants and their families.

## Funding

This work was supported by the National Natural Science Foundation of China (Grants 62373056 and 62206165), and the Clinical Research Program and Brain–Computer Interface (BCI) Research Program of 9th People’s Hospital, Shanghai Jiao Tong University School of Medicine.

## Disclosure

The authors take full responsibility for the data, the analyses and interpretation, and the conduct of the research. The authors had full access to all of the data and had the right to publish any and all the data separately and apart from any sponsor.

## Ethics Statement

This study received approval from the Ethics Committee of Shanghai Jiao Tong University (Approval Number 2017‐365‐T267). The study was registered in the Chinese Clinical Trial Registry (Registration Number ChiCTR1800014630).

## Consent

Written informed consent was obtained from all volunteers and patients prior to participation.

## Conflicts of Interest

The authors declare no conflicts of interest.

## Supporting Information

Additional supporting information can be found online in the Supporting Information section.

## Supporting information


**Supporting Information** Table S1, the STROBE Checklist for Observational Studies, is provided to document the reporting guidelines followed in this study. In addition, supplementary materials include the head‐motion exclusion details for all participants across the six motor tasks. Tables S2 and S3 provide the detailed head‐motion metrics for the NC and FS groups, respectively. Table S4 summarizes the numbers and group distributions of participants excluded for excessive head motion across the six motor tasks.

## Data Availability

The data that support the findings of this study are available upon reasonable request.

## References

[1] Longino E. S. , Desisto N. G. , and Ortiz A. S. , et al.Effect of Underlying Mental Health Disorders on the Correlation Between Patient- and Surgeon-Graded Synkinesis Scores., Facial Plastic Surgery & Aesthetic Medicine. (2024) 26, no. 5, 544–550, 10.1089/fpsam.2023.0291. 38569157

[2] Krane N. A. , Loyo M. , Pollock J. , Hill M. , Johnson C. Z. , and Stevens A. A. , Exploratory Study of the Brain Response in Facial Synkinesis After Bell Palsy With Systematic Review and Meta-Analysis of the Literature, American Journal of Neuroradiology. (2022) 43, no. 10, 1470–1475, 10.3174/ajnr.A7619. 36574328 PMC9575525

[3] Husseman J. and Mehta R. P. , Management of Synkinesis. Facial Plast Surg, Facial Plastic Surgery. (2008) 24, no. 2, 242–249, 10.1055/s-2008-1075840, 2-s2.0-44149112356. 18470836

[4] Fujiwara K. , Fukuda A. , and Morita S. , et al.Psychological Evaluation for Patients With Non-Cured Facial Nerve Palsy, Auris Nasus Larynx. (2022) 49, no. 1, 53–57, 10.1016/j.anl.2021.04.007. 33962818

[5] Martino S. C. , Elliott M. N. , Kanouse D. E. , Farley D. O. , Burkhart Q. , and Hays R. D. , Depression and the Health Care Experiences of Medicare Beneficiaries, Health Services Research. (2011) 46, no. 6pt1, 1883–1904, 10.1111/j.1475-6773.2011.01293.x, 2-s2.0-81755179402. 21762146 PMC3197881

[6] Ekpo E. , Beynel L. , and Deng Z. , et al.227. Goal Priming: Using a Task to Assess Functional Connectivity in Depression, Biological Psychiatry. (2024) 95, no. 10, S192–S193, 10.1016/j.biopsych.2024.02.462.

[7] Zhang J.-X. , Bo K. , Wager T. D. , and Gross J. J. , The Brain Bases of Emotion Generation and Emotion Regulation, Trends in Cognitive Sciences. (2025) 29, no. 10, 879–891, 10.1016/j.tics.2025.04.013. 40447491

[8] Goldin P. R. , McRae K. , Ramel W. , and Gross J. J. , The Neural Bases of Emotion Regulation: Reappraisal and Suppression of Negative Emotion, Biological Psychiatry. (2008) 63, no. 6, 577–586, 10.1016/j.biopsych.2007.05.031, 2-s2.0-39449123341. 17888411 PMC2483789

[9] Roberts A. C. and Mulvihill K. G. , Multiple Faces of Anxiety: A Frontal Lobe Perspective, Trends in Neurosciences. (2024) 47, no. 9, 708–721, 10.1016/j.tins.2024.07.001. 39127569

[10] Dijkstra E. S. A. , Frandsen S. B. , and van Dijk H. , et al.Probing Prefrontal-SgACC Connectivity Using TMS-Induced Heart–Brain Coupling, Nature Mental Health. (2024) 2, no. 7, 809–817, 10.1038/s44220-024-00248-8.

[11] Qiao D. , Li Y. , and Zhang X. , et al.Exploring the Connectivity of Dorsolateral Prefrontal Cortex and the Modulatory Impact of Transcranial Magnetic Stimulation in Adolescents With Depression: A Focus on Pain-Related Cognitive Processing, BMC Psychiatry. (2024) 24, no. 1, 10.1186/s12888-024-06321-x, 852. 39604966 PMC11600770

[12] Colich N. L. , Ho T. C. , and Foland-Ross L. C. , et al.Hyperactivation in Cognitive Control and Visual Attention Brain Regions During Emotional Interference in Adolescent Depression, Biological Psychiatry: Cognitive Neuroscience and Neuroimaging. (2017) 2, no. 5, 388–395, 10.1016/j.bpsc.2016.09.001, 2-s2.0-85006778772. 28890942 PMC5586219

[13] Zhang B. , Qi S. , Liu S. , Liu X. , Wei X. , and Ming D. , Altered Spontaneous Neural Activity in the Precuneus, Middle and Superior Frontal Gyri, and Hippocampus in College Students With Subclinical Depression, BMC Psychiatry. (2021) 21, no. 1, 10.1186/s12888-021-03292-1, 280. 34074266 PMC8167968

[14] Zhang S. , Zhang Y. , Ma W. , Qi Z. , Wang Y. , and Tao Q. , Neural Correlates of Negative Emotion Processing in Subthreshold Depression., Social Cognitive and Affective Neuroscience. (2022) 17, no. 7, 655–661, 10.1093/scan/nsac003. 35156124 PMC9250298

[15] Wang Y. , Wang W.-W. , Hua X.-Y. , Liu H.-Q. , and Ding W. , Patterns of Cortical Reorganization in Facial Synkinesis: A Task Functional Magnetic Resonance Imaging Study, Neural Regeneration Research. (2018) 13, no. 9, 1637–1642, 10.4103/1673-5374.235304, 2-s2.0-85051955790. 30127126 PMC6126138

[16] Zhang C.-H. , Wang H.-Q. , Lu Y. , Wang W. , Ma H. , and Lu Y.-C. , Exploration of Rich-Club Reorganization in Facial Synkinesis: Insights From Structural and Functional Brain Network Analysis, Cerebral Cortex. (2023) 33, no. 24, 11570–11581, 10.1093/cercor/bhad390. 37851710

[17] Guntinas-Lichius O. , Prengel J. , and Cohen O. , et al.Pathogenesis, Diagnosis and Therapy of Facial Synkinesis: A Systematic Review and Clinical Practice Recommendations by the International Head and Neck Scientific Group, Frontiers in Neurology. (2022) 13, 10.3389/fneur.2022.1019554, 1019554. 36438936 PMC9682287

[18] Deligianni F. , Guo Y. , and Yang G.-Z. , From Emotions to Mood Disorders: A Survey on Gait Analysis Methodology, IEEE Journal of Biomedical and Health Informatics. (2019) 23, no. 6, 2302–2316, 10.1109/JBHI.2019.2938111. 31502995

[19] Kebets V. , Holmes A. J. , and Orban C. , et al.Somatosensory-Motor Dysconnectivity Spans Multiple Transdiagnostic Dimensions of Psychopathology, Biological Psychiatry. (2019) 86, no. 10, 779–791, 10.1016/j.biopsych.2019.06.013, 2-s2.0-85071849525. 31515054

[20] von Glischinski M. , von Brachel R. , and Hirschfeld G. , How Depressed is “Depressed"? A Systematic Review and Diagnostic Meta-Analysis of Optimal Cut Points for the Beck Depression Inventory Revised (BDI-II), Quality of Life Research. (2019) 28, no. 5, 1111–1118, 10.1007/s11136-018-2050-x, 2-s2.0-85056812839. 30456716

[21] Wang L. , Li C. , and Han Z. , et al.Spatiotemporal and Sensory Modality Attention Processing With Domain-Specific Representations in Frontoparietal Areas, Cerebral Cortex. (2022) 32, no. 24, 5489–5502, 10.1093/cercor/bhac029. 35136999

[22] Tzourio-Mazoyer N. , Landeau B. , and Papathanassiou D. , et al.Automated Anatomical Labeling of Activations in SPM Using a Macroscopic Anatomical Parcellation of the MNI MRI Single-Subject Brain, NeuroImage. (2002) 15, no. 1, 273–289, 10.1006/nimg.2001.0978, 2-s2.0-0036322886. 11771995

[23] Sala-Llonch R. , Smith S. M. , and Woolrich M. , et al.Spatial Parcellations, Spectral Filtering, and Connectivity Measures in fMRI: Optimizing for Discrimination, Human Brain Mapping. (2019) 40, no. 2, 407–419, 10.1002/hbm.24381, 2-s2.0-85053887222. 30259597 PMC6492132

[24] Rostami S. , Min S. , and McCann A. , et al.The Effectiveness of Facial Neuromuscular Retraining on Patients With Facial Nerve Dysfunction: A Mental Health and Quality of Life Analysis, Facial Plastic Surgery & Aesthetic Medicine. (2024) 26, no. 5, 551–557, 10.1089/fpsam.2023.0119. 38635958

[25] Ma J. , Hua X. Y. , and Zheng M. X. , et al.Spatial Patterns of Intrinsic Brain Activity and Functional Connectivity in Facial Synkinesis Patients, British Journal of Neurosurgery. (2021) 35, no. 6, 730–735, 10.1080/02688697.2020.1773396. 32500814

[26] Wu J. J. , Lu Y. C. , and Zheng M. X. , et al.Structural Remodeling in Related Brain Regions in Patients With Facial Synkinesis, Neural Regeneration Research. (2021) 16, no. 12, 2528–2533, 10.4103/1673-5374.313055. 33907044 PMC8374555

[27] Smit A. , van der Geest J. , Metselaar M. , van der Lugt A. , VanderWerf F. , and De Zeeuw C. , Long-Term Changes in Cerebellar Activation During Functional Recovery From Transient Peripheral Motor Paralysis, Experimental Neurology. (2010) 226, no. 1, 33–39, 10.1016/j.expneurol.2010.07.026, 2-s2.0-77957857904. 20691681

[28] Wang Y. , Yang L. , Wang W.-W. , Ding W. , and Liu H.-Q. , Decreased Distance Between Representation Sites of Distinct Facial Movements in Facial Synkinesis—A Task fMRI Study, Neuroscience. (2019) 397, 12–17, 10.1016/j.neuroscience.2018.11.036, 2-s2.0-85057854041. 30500612

[29] Bitter T. , Sorger B. , Hesselmann V. , Krug B. , Lackner K. , and Guntinas-Lichius O. , Cortical Representation Sites of Mimic Movements after Facial Nerve Reconstruction: A Functional Magnetic Resonance Imaging Study, The Laryngoscope. (2011) 121, no. 4, 699–706, 10.1002/lary.21399, 2-s2.0-79953093889. 21287559

[30] Zagha E. , Casale A E. , Sachdev R N. S. , McGinley M J. , and McCormick D A. , Motor Cortex Feedback Influences Sensory Processing by Modulating Network State, Neuron. (2013) 79, no. 3, 567–578, 10.1016/j.neuron.2013.06.008, 2-s2.0-84881496012. 23850595 PMC3742632

[31] Whyte C. J. , Redinbaugh M. J. , Shine J. M. , and Saalmann Y. B. , Thalamic Contributions to the State and Contents of Consciousness, Neuron. (2024) 112, no. 10, 1611–1625, 10.1016/j.neuron.2024.04.019. 38754373 PMC11537458

[32] Fang Z. , Dang Y. , and Ping A. , et al.Human High-Order Thalamic Nuclei Gate Conscious Perception Through the Thalamofrontal Loop, Science. (2025) 388, no. 6742, 10.1126/science.adr3675. 40179184

[33] Alagapan S. , Choi K. S. , and Heisig S. , et al.Cingulate Dynamics Track Depression Recovery With Deep Brain Stimulation, Nature. (2023) 622, no. 7981, 130–138, 10.1038/s41586-023-06541-3. 37730990 PMC10550829

[34] Xiao J. , Adkinson J. A. , and Myers J. , et al.Beta Activity in Human Anterior Cingulate Cortex Mediates Reward Biases, Nature Communications. (2024) 15, no. 1, 10.1038/s41467-024-49600-7, 5528. PMC11250824 39009561

[35] Cavanagh J. F. and Frank M. J. , Frontal Theta as a Mechanism for Cognitive Control, Trends in Cognitive Sciences. (2014) 18, no. 8, 414–421, 10.1016/j.tics.2014.04.012, 2-s2.0-84904718962. 24835663 PMC4112145

[36] Ebneabbasi A. , Mahdipour M. , and Nejati V. , et al.Emotion Processing and Regulation in Major Depressive Disorder: A 7T Resting-State fMRI Study, Human Brain Mapping. (2021) 42, no. 3, 797–810, 10.1002/hbm.25263. 33151031 PMC7814754

[37] Picó-Pérez M. , Radua J. , Steward T. , Menchón Jé M. , and Soriano-Mas C. , Emotion Regulation in Mood and Anxiety Disorders: A Meta-Analysis of fMRI Cognitive Reappraisal Studies, Progress in Neuro-Psychopharmacology and Biological Psychiatry. (2017) 79, no. Pt B, 96–104, 10.1016/j.pnpbp.2017.06.001, 2-s2.0-85021167708. 28579400

[38] Leone C. , Feys P. , Moumdjian L. , D’Amico E. , Zappia M. , and Patti F. , Cognitive-Motor Dual-Task Interference: A Systematic Review of Neural Correlates, Neuroscience & Biobehavioral Reviews. (2017) 75, 348–360, 10.1016/j.neubiorev.2017.01.010, 2-s2.0-85013200641. 28104413

[39] Coulson S. E. , O’Dwyer N. J. , Adams R. D. , and Croxson G. R. , Expression of Emotion and Quality of Life After Facial Nerve Paralysis, Otology & Neurotology. (2004) 25, no. 6, 1014–1019, 10.1097/00129492-200411000-00026, 2-s2.0-8544221929. 15547436

[40] Jowett N. , Malka R. , and Hadlock T. A. , Effect of Weakening of Ipsilateral Depressor Anguli Oris on Smile Symmetry in Postparalysis Facial Palsy, JAMA Facial Plastic Surgery. (2017) 19, no. 1, 29–33, 10.1001/jamafacial.2016.1115, 2-s2.0-85012895739. 27658020

[41] O’Rourke S. P. , Stack T. J. , Miller J. R. , and Miller M. Q. , Changes in Perceived Emotions in Facial Paralysis Patients After Depressor Anguli Oris Excision, The Laryngoscope. (2024) 134, no. 9, 4028–4035, 10.1002/lary.31471. 38706403

[42] Fearington F. W. , Rodriguez G. , Randall N. R. , and Dey J. K. , Surgical Treatments for Facial Aberrant Reinnervation Syndrome: A Systematic Review. Facial Plast Surg Aesthet Med, Facial Plastic Surgery & Aesthetic Medicine. (2025) 27, no. 4, 330–343, 10.1089/fpsam.2024.0147. 39505694

[43] He Z. , Yang K. , Zhuang N. , and Zeng Y. , Processing of Affective Pictures: A Study Based on Functional Connectivity Network in the Cerebral Cortex, Computational Intelligence and Neuroscience. (2021) 2021, no. 1, 10.1155/2021/5582666, 5582666. 34257637 PMC8245225

[44] Sun S. , Yu H. , Yu R. , and Wang S. , Functional Connectivity Between the Amygdala and Prefrontal Cortex Underlies Processing of Emotion Ambiguity, Translational Psychiatry. (2023) 13, no. 1, 10.1038/s41398-023-02625-w, 334. 37898626 PMC10613296

[45] Lutz J. , Herwig U. , and Opialla S. , et al.Mindfulness and Emotion Regulation—an fMRI Study, Social Cognitive and Affective Neuroscience. (2014) 9, no. 6, 776–785, 10.1093/scan/nst043, 2-s2.0-84902251002. 23563850 PMC4040090

[46] Zhang Z. , Zhang Y. , and Wang H. , et al.Resting-State Network Alterations in Depression: A Comprehensive Meta-Analysis of Functional Connectivity, Psychological Medicine. (2025) 55, 10.1017/S0033291725000303. PMC12080655 40008424

[47] Li W. , Wang C. , and Lan X. , et al.Resting-State Functional Connectivity of the Amygdala in Major Depressive Disorder With Suicidal Ideation, Journal of Psychiatric Research. (2022) 153, 189–196, 10.1016/j.jpsychires.2022.07.001. 35839660

[48] Prentice A. , Kolken Y. , and Tuttle C. , et al.1Hz Right Orbitofrontal TMS Benefits Depressed Patients Unresponsive to Dorsolateral Prefrontal Cortex TMS, Brain Stimulation. (2023) 16, no. 6, 1572–1575, 10.1016/j.brs.2023.10.005. 37839775

[49] Morriss R. , Briley P. M. , and Webster L. , et al.Connectivity-Guided Intermittent Theta Burst Versus Repetitive Transcranial Magnetic Stimulation for Treatment-Resistant Depression: A Randomized Controlled Trial, Nature Medicine. (2024) 30, no. 2, 403–413, 10.1038/s41591-023-02764-z. PMC10878976 38228914

