# Supplementary material for: Abnormal Activation and Connection in Middle Frontal Gyrus: A Potential Imaging Feature for Facial Synkinesis Comorbid Depression
Source: Depress Anxiety. 2026 Apr 25;2026:1705507. doi: 10.1155/da/1705507 (PMC13110137; doi:10.1155/da/1705507)
Supplement: Supplementary file 1 — Supporting Information Table S1, the STROBE Checklist for Observational Studies, is provided to document the reporting guidelines followed in this study. In addition, supplementary materials include the head‐motion exclusion details for all participants across the six motor tasks. Tables S2 and S3 provide the detailed head‐motion metrics for the NC and FS groups, respectively. Table S4 summarizes the numbers and group distributions of participants excluded for excessive head motion across the six motor tasks. [file DA-2026-1705507-s001.docx]

Sup Table 2. NC group motion.

| Subjects | Task | Mean Tran (mm) | Max Tran  (mm) | Mean Rot  (deg) | Max Rot  (deg) |
| --- | --- | --- | --- | --- | --- |
| sub001 | Left Blink Motor | 0.40 | 0.61 | 0.01 | 0.02 |
|  | Left Grin Motor | 0.22 | 0.38 | 0.01 | 0.01 |
|  | Left Smile Motor | 0.09 | 0.14 | 0.00 | 0.01 |
|  | Right Blink Motor | 0.27 | 0.55 | 0.01 | 0.01 |
|  | Right Grin Motor | 0.30 | 0.46 | 0.02 | 0.03 |
|  | Right Smile Motor | 0.51 | 0.91 | 0.01 | 0.02 |
| sub002 | Left Blink Motor | 0.07 | 0.14 | 0.00 | 0.00 |
|  | Left Grin Motor | 0.13 | 0.27 | 0.01 | 0.02 |
|  | Left Smile Motor | 0.11 | 0.18 | 0.01 | 0.02 |
|  | Right Blink Motor | 0.07 | 0.18 | 0.01 | 0.02 |
|  | Right Grin Motor | 0.19 | 0.31 | 0.01 | 0.01 |
|  | Right Smile Motor | 0.08 | 0.13 | 0.00 | 0.00 |
| sub003 | Left Blink Motor | 0.45 | 0.77 | 0.01 | 0.02 |
|  | Left Grin Motor | 0.29 | 1.18 | 0.01 | 0.02 |
|  | Left Smile Motor | 0.12 | 0.22 | 0.00 | 0.01 |
|  | Right Blink Motor | 0.80 | 1.25 | 0.01 | 0.02 |
|  | Right Grin Motor | 0.62 | 1.06 | 0.01 | 0.01 |
|  | Right Smile Motor | 0.26 | 1.46 | 0.01 | 0.02 |
| sub004 | Left Blink Motor | 0.19 | 0.31 | 0.01 | 0.02 |
|  | Left Grin Motor | 0.16 | 0.30 | 0.01 | 0.02 |
|  | Left Smile Motor | 0.16 | 0.30 | 0.01 | 0.01 |
|  | Right Blink Motor | 0.16 | 0.29 | 0.01 | 0.01 |
|  | Right Grin Motor | 0.21 | 0.34 | 0.01 | 0.01 |
|  | Right Smile Motor | 0.36 | 0.49 | 0.01 | 0.02 |
| sub005 | Left Blink Motor | 0.11 | 0.18 | 0.00 | 0.00 |
|  | Left Grin Motor | 0.10 | 0.25 | 0.01 | 0.01 |
|  | Left Smile Motor | 0.05 | 0.11 | 0.00 | 0.00 |
|  | Right Blink Motor | 0.09 | 0.16 | 0.01 | 0.01 |
|  | Right Grin Motor | 0.09 | 0.19 | 0.00 | 0.01 |
|  | Right Smile Motor | 0.11 | 0.18 | 0.01 | 0.01 |
| sub006 | Left Blink Motor | 0.07 | 0.16 | 0.00 | 0.01 |
|  | Left Grin Motor | 0.10 | 0.21 | 0.01 | 0.01 |
|  | Left Smile Motor | 0.13 | 0.22 | 0.01 | 0.01 |
|  | Right Blink Motor | 0.10 | 0.26 | 0.01 | 0.01 |
|  | Right Grin Motor | 0.15 | 0.38 | 0.01 | 0.02 |
|  | Right Smile Motor | 0.25 | 0.42 | 0.02 | 0.02 |
| sub007 | Left Blink Motor | 0.10 | 0.29 | 0.00 | 0.00 |
|  | Left Grin Motor | 0.21 | 0.38 | 0.01 | 0.02 |
|  | Left Smile Motor | 0.27 | 0.64 | 0.01 | 0.01 |
|  | Right Blink Motor | 0.39 | 1.01 | 0.01 | 0.01 |
|  | Right Grin Motor | 0.31 | 0.59 | 0.01 | 0.01 |
|  | Right Smile Motor | 0.27 | 0.42 | 0.00 | 0.01 |
| sub008 | Left Blink Motor | 0.10 | 0.17 | 0.01 | 0.01 |
|  | Left Grin Motor | 0.10 | 0.17 | 0.00 | 0.00 |
|  | Left Smile Motor | 0.18 | 0.37 | 0.00 | 0.01 |
|  | Right Blink Motor | 0.09 | 0.14 | 0.01 | 0.01 |
|  | Right Grin Motor | 0.11 | 0.19 | 0.00 | 0.01 |
|  | Right Smile Motor | 0.69 | 1.62 | 0.01 | 0.01 |
| sub009 | Left Blink Motor | 0.16 | 0.28 | 0.01 | 0.02 |
|  | Left Grin Motor | 0.34 | 0.61 | 0.01 | 0.02 |
|  | Left Smile Motor | 0.21 | 0.40 | 0.00 | 0.00 |
|  | Right Blink Motor | 0.41 | 0.61 | 0.00 | 0.01 |
|  | Right Grin Motor | 0.33 | 0.55 | 0.02 | 0.03 |
|  | Right Smile Motor | 0.16 | 0.26 | 0.01 | 0.01 |
| sub010 | Left Blink Motor | 0.36 | 0.58 | 0.01 | 0.01 |
|  | Left Grin Motor | 0.57 | 0.96 | 0.02 | 0.03 |
|  | Left Smile Motor | 0.23 | 0.52 | 0.01 | 0.01 |
|  | Right Blink Motor | 0.41 | 0.79 | 0.01 | 0.02 |
|  | Right Grin Motor | 0.26 | 0.51 | 0.01 | 0.01 |
|  | Right Smile Motor | 0.21 | 0.33 | 0.00 | 0.01 |
| sub011 | Left Blink Motor | 0.76 | 0.98 | 0.03 | 0.04 |
|  | Left Grin Motor | 0.69 | 1.22 | 0.02 | 0.03 |
|  | Left Smile Motor | 0.82 | 1.17 | 0.02 | 0.02 |
|  | Right Blink Motor | 0.45 | 0.78 | 0.02 | 0.04 |
|  | Right Grin Motor | 0.24 | 0.45 | 0.01 | 0.02 |
|  | Right Smile Motor | 0.49 | 0.78 | 0.02 | 0.02 |
| sub012 | Left Blink Motor | 0.76 | 0.98 | 0.03 | 0.04 |
|  | Left Grin Motor | 0.69 | 1.22 | 0.02 | 0.03 |
|  | Left Smile Motor | 0.82 | 1.17 | 0.02 | 0.02 |
|  | Right Blink Motor | 0.45 | 0.78 | 0.02 | 0.04 |
|  | Right Grin Motor | 0.24 | 0.45 | 0.01 | 0.02 |
|  | Right Smile Motor | 0.49 | 0.78 | 0.02 | 0.02 |
| sub013 | Left Blink Motor | 0.06 | 0.14 | 0.01 | 0.01 |
|  | Left Grin Motor | 0.37 | 0.69 | 0.01 | 0.02 |
|  | Left Smile Motor | 0.20 | 0.34 | 0.02 | 0.03 |
|  | Right Blink Motor | 0.40 | 0.74 | 0.02 | 0.03 |
|  | Right Grin Motor | 0.12 | 0.34 | 0.01 | 0.01 |
|  | Right Smile Motor | 0.16 | 0.31 | 0.01 | 0.01 |
| sub014 | Left Blink Motor | 0.33 | 1.03 | 0.01 | 0.01 |
|  | Left Grin Motor | 0.33 | 0.55 | 0.01 | 0.01 |
|  | Left Smile Motor | 0.61 | 1.16 | 0.01 | 0.01 |
|  | Right Blink Motor | 0.17 | 0.38 | 0.01 | 0.02 |
|  | Right Grin Motor | 0.66 | 1.20 | 0.01 | 0.02 |
|  | Right Smile Motor | 0.92 | 1.83 | 0.02 | 0.04 |
| sub015 | Left Blink Motor | 0.33 | 1.03 | 0.01 | 0.01 |
|  | Left Grin Motor | 0.33 | 0.55 | 0.01 | 0.01 |
|  | Left Smile Motor | 0.61 | 1.16 | 0.01 | 0.01 |
|  | Right Blink Motor | 0.17 | 0.38 | 0.01 | 0.02 |
|  | Right Grin Motor | 0.66 | 1.20 | 0.01 | 0.02 |
|  | Right Smile Motor | 0.92 | 1.83 | 0.02 | 0.04 |
| sub016 | Left Blink Motor | 0.15 | 0.28 | 0.01 | 0.01 |
|  | Left Grin Motor | 0.08 | 0.16 | 0.00 | 0.01 |
|  | Left Smile Motor | 0.10 | 0.16 | 0.00 | 0.00 |
|  | Right Blink Motor | 0.21 | 0.35 | 0.01 | 0.01 |
|  | Right Grin Motor | 0.20 | 0.46 | 0.01 | 0.01 |
|  | Right Smile Motor | 0.05 | 0.13 | 0.00 | 0.01 |
| sub017 | Left Blink Motor | 0.15 | 0.28 | 0.01 | 0.01 |
|  | Left Grin Motor | 0.08 | 0.16 | 0.00 | 0.01 |
|  | Left Smile Motor | 0.10 | 0.16 | 0.00 | 0.00 |
|  | Right Blink Motor | 0.21 | 0.35 | 0.01 | 0.01 |
|  | Right Grin Motor | 0.20 | 0.46 | 0.01 | 0.01 |
|  | Right Smile Motor | 0.05 | 0.13 | 0.00 | 0.01 |
| sub018 | Left Blink Motor | 0.25 | 0.59 | 0.01 | 0.01 |
|  | Left Grin Motor | 0.67 | 1.26 | 0.01 | 0.02 |
|  | Left Smile Motor | 0.22 | 0.36 | 0.01 | 0.01 |
|  | Right Blink Motor | 0.14 | 0.24 | 0.01 | 0.01 |
|  | Right Grin Motor | 0.56 | 1.01 | 0.01 | 0.02 |
|  | Right Smile Motor | 0.41 | 0.55 | 0.01 | 0.01 |
| sub019 | Left Blink Motor | 0.30 | 0.48 | 0.00 | 0.00 |
|  | Left Grin Motor | 0.53 | 0.99 | 0.03 | 0.05 |
|  | Left Smile Motor | 0.84 | 1.29 | 0.03 | 0.05 |
|  | Right Blink Motor | 1.36 | 1.79 | 0.03 | 0.03 |
|  | Right Grin Motor | 0.62 | 1.07 | 0.02 | 0.03 |
|  | Right Smile Motor | 0.29 | 0.90 | 0.01 | 0.02 |

Sup Table 3. FS group motion.

| Subjects | Task | Mean Tran  (mm) | Max Tran  (mm) | Mean Rot  (deg) | Max Rot  (deg) |
| --- | --- | --- | --- | --- | --- |
| sub021 | Left Blink Motor | 0.16 | 0.28 | 0.01 | 0.03 |
|  | Left Grin Motor | 0.88 | 1.39 | 0.02 | 0.04 |
|  | Left Smile Motor | 0.24 | 0.43 | 0.01 | 0.02 |
|  | Right Blink Motor | 0.29 | 0.53 | 0.01 | 0.02 |
|  | Right Grin Motor | 0.46 | 1.08 | 0.02 | 0.03 |
|  | Right Smile Motor | 0.37 | 0.59 | 0.01 | 0.02 |
| sub022 | Left Blink Motor | 0.09 | 0.17 | 0.00 | 0.00 |
|  | Left Grin Motor | 0.30 | 0.56 | 0.01 | 0.02 |
|  | Left Smile Motor | 0.25 | 0.40 | 0.00 | 0.01 |
|  | Right Blink Motor | 0.11 | 0.29 | 0.01 | 0.01 |
|  | Right Grin Motor | 0.19 | 0.35 | 0.01 | 0.01 |
|  | Right Smile Motor | 0.27 | 0.44 | 0.01 | 0.02 |
| sub023 | Left Blink Motor | 0.09 | 0.17 | 0.00 | 0.00 |
|  | Left Grin Motor | 0.30 | 0.56 | 0.01 | 0.02 |
|  | Left Smile Motor | 0.25 | 0.40 | 0.00 | 0.01 |
|  | Right Blink Motor | 0.11 | 0.29 | 0.01 | 0.01 |
|  | Right Grin Motor | 0.19 | 0.35 | 0.01 | 0.01 |
|  | Right Smile Motor | 0.27 | 0.44 | 0.01 | 0.02 |
| sub024 | Left Blink Motor | 0.24 | 0.38 | 0.01 | 0.02 |
|  | Left Grin Motor | 0.12 | 0.36 | 0.00 | 0.01 |
|  | Left Smile Motor | 0.37 | 0.58 | 0.01 | 0.01 |
|  | Right Blink Motor | 0.43 | 0.92 | 0.03 | 0.04 |
|  | Right Grin Motor | 0.22 | 0.49 | 0.01 | 0.01 |
|  | Right Smile Motor | 0.51 | 0.83 | 0.01 | 0.03 |
| sub025 | Left Blink Motor | 0.12 | 0.29 | 0.01 | 0.01 |
|  | Left Grin Motor | 0.43 | 0.68 | 0.01 | 0.02 |
|  | Left Smile Motor | 0.27 | 0.50 | 0.00 | 0.01 |
|  | Right Blink Motor | 0.10 | 0.19 | 0.00 | 0.01 |
|  | Right Grin Motor | 0.14 | 0.22 | 0.01 | 0.01 |
|  | Right Smile Motor | 0.49 | 0.78 | 0.02 | 0.03 |
| sub026 | Left Blink Motor | 0.17 | 0.29 | 0.01 | 0.01 |
|  | Left Grin Motor | 0.16 | 0.27 | 0.01 | 0.02 |
|  | Left Smile Motor | 0.16 | 0.24 | 0.01 | 0.01 |
|  | Right Blink Motor | 0.33 | 0.51 | 0.02 | 0.03 |
|  | Right Grin Motor | 0.16 | 0.30 | 0.01 | 0.01 |
|  | Right Smile Motor | 0.30 | 0.55 | 0.00 | 0.01 |
| sub027 | Left Blink Motor | 0.75 | 2.11 | 0.02 | 0.05 |
|  | Left Grin Motor | 0.44 | 0.81 | 0.01 | 0.02 |
|  | Left Smile Motor | 0.99 | 1.40 | 0.02 | 0.03 |
|  | Right Blink Motor | 0.86 | 1.80 | 0.02 | 0.03 |
|  | Right Grin Motor | 0.51 | 0.79 | 0.01 | 0.02 |
|  | Right Smile Motor | 1.10 | 1.70 | 0.02 | 0.04 |
| sub028 | Left Blink Motor | 0.17 | 0.31 | 0.00 | 0.01 |
|  | Left Grin Motor | 0.46 | 1.07 | 0.01 | 0.03 |
|  | Left Smile Motor | 0.16 | 0.31 | 0.01 | 0.01 |
|  | Right Blink Motor | 0.28 | 0.46 | 0.01 | 0.02 |
|  | Right Grin Motor | 0.24 | 0.39 | 0.01 | 0.01 |
|  | Right Smile Motor | 0.15 | 0.40 | 0.00 | 0.01 |
| sub029 | Left Blink Motor | 0.40 | 0.83 | 0.02 | 0.04 |
|  | Left Grin Motor | 0.19 | 0.39 | 0.01 | 0.01 |
|  | Left Smile Motor | 0.54 | 0.95 | 0.02 | 0.05 |
|  | Right Blink Motor | 0.28 | 0.61 | 0.01 | 0.01 |
|  | Right Grin Motor | 0.31 | 0.62 | 0.01 | 0.01 |
|  | Right Smile Motor | 0.09 | 0.19 | 0.00 | 0.01 |
| sub030 | Left Blink Motor | 0.22 | 0.40 | 0.02 | 0.04 |
|  | Left Grin Motor | 0.42 | 0.61 | 0.01 | 0.02 |
|  | Left Smile Motor | 0.24 | 0.41 | 0.03 | 0.05 |
|  | Right Blink Motor | 0.10 | 0.33 | 0.01 | 0.01 |
|  | Right Grin Motor | 0.31 | 0.48 | 0.01 | 0.01 |
|  | Right Smile Motor | 0.30 | 0.67 | 0.02 | 0.03 |
| sub031 | Left Blink Motor | 0.19 | 0.41 | 0.01 | 0.01 |
|  | Left Grin Motor | 0.22 | 0.41 | 0.01 | 0.01 |
|  | Left Smile Motor | 0.14 | 0.38 | 0.01 | 0.01 |
|  | Right Blink Motor | 0.29 | 0.47 | 0.03 | 0.05 |
|  | Right Grin Motor | 0.18 | 0.30 | 0.01 | 0.01 |
|  | Right Smile Motor | 0.30 | 0.45 | 0.01 | 0.01 |
| sub032 | Left Blink Motor | 0.48 | 0.70 | 0.02 | 0.03 |
|  | Left Grin Motor | 0.60 | 1.05 | 0.02 | 0.03 |
|  | Left Smile Motor | 0.45 | 0.79 | 0.01 | 0.03 |
|  | Right Blink Motor | 0.25 | 0.49 | 0.01 | 0.05 |
|  | Right Grin Motor | 0.40 | 0.55 | 0.02 | 0.03 |
|  | Right Smile Motor | 0.13 | 0.19 | 0.01 | 0.01 |
| sub033 | Left Blink Motor | 0.73 | 1.13 | 0.04 | 0.07 |
|  | Left Grin Motor | 1.68 | 2.83 | 0.04 | 0.08 |
|  | Left Smile Motor | 0.90 | 1.49 | 0.04 | 0.05 |
|  | Right Blink Motor | 0.52 | 0.76 | 0.04 | 0.06 |
|  | Right Grin Motor | 1.29 | 2.64 | 0.04 | 0.08 |
|  | Right Smile Motor | 0.91 | 1.32 | 0.04 | 0.05 |
| sub034 | Left Blink Motor | 0.12 | 0.47 | 0.01 | 0.02 |
|  | Left Grin Motor | 0.29 | 0.52 | 0.01 | 0.02 |
|  | Left Smile Motor | 0.12 | 0.25 | 0.01 | 0.01 |
|  | Right Blink Motor | 0.22 | 0.33 | 0.01 | 0.01 |
|  | Right Grin Motor | 0.21 | 0.33 | 0.01 | 0.02 |
|  | Right Smile Motor | 0.25 | 0.35 | 0.01 | 0.01 |
| sub035 | Left Blink Motor | 0.26 | 0.51 | 0.02 | 0.03 |
|  | Left Grin Motor | 0.47 | 0.75 | 0.03 | 0.05 |
|  | Left Smile Motor | 0.11 | 0.20 | 0.01 | 0.02 |
|  | Right Blink Motor | 0.11 | 0.22 | 0.01 | 0.01 |
|  | Right Grin Motor | 0.33 | 0.56 | 0.01 | 0.01 |
|  | Right Smile Motor | 0.25 | 0.38 | 0.02 | 0.02 |
| sub036 | Left Blink Motor | 0.10 | 0.26 | 0.00 | 0.01 |
|  | Left Grin Motor | 0.21 | 0.48 | 0.00 | 0.02 |
|  | Left Smile Motor | 0.08 | 0.16 | 0.00 | 0.01 |
|  | Right Blink Motor | 0.34 | 0.76 | 0.01 | 0.01 |
|  | Right Grin Motor | 0.20 | 0.57 | 0.00 | 0.00 |
|  | Right Smile Motor | 0.09 | 0.16 | 0.00 | 0.01 |
| sub037 | Left Blink Motor | 0.19 | 0.47 | 0.01 | 0.01 |
|  | Left Grin Motor | 0.33 | 0.55 | 0.01 | 0.02 |
|  | Left Smile Motor | 0.46 | 0.78 | 0.01 | 0.02 |
|  | Right Blink Motor | 0.43 | 0.67 | 0.01 | 0.02 |
|  | Right Grin Motor | 0.58 | 1.35 | 0.02 | 0.03 |
|  | Right Smile Motor | 0.48 | 0.97 | 0.01 | 0.02 |
| sub038 | Left Blink Motor | 0.27 | 0.49 | 0.01 | 0.02 |
|  | Left Grin Motor | 0.96 | 1.84 | 0.02 | 0.04 |
|  | Left Smile Motor | 1.13 | 1.74 | 0.01 | 0.02 |
|  | Right Blink Motor | 0.20 | 0.65 | 0.01 | 0.02 |
|  | Right Grin Motor | 1.40 | 3.03 | 0.03 | 0.08 |
|  | Right Smile Motor | 0.30 | 0.55 | 0.01 | 0.01 |
| sub039 | Left Blink Motor | 0.23 | 0.64 | 0.03 | 0.04 |
|  | Left Grin Motor | 0.55 | 0.94 | 0.02 | 0.04 |
|  | Left Smile Motor | 0.18 | 0.45 | 0.01 | 0.02 |
|  | Right Blink Motor | 1.13 | 2.63 | 0.03 | 0.04 |
|  | Right Grin Motor | 0.18 | 0.34 | 0.01 | 0.02 |
|  | Right Smile Motor | 0.13 | 0.26 | 0.01 | 0.01 |
| sub040 | Left Blink Motor | 0.13 | 0.39 | 0.00 | 0.01 |
|  | Left Grin Motor | 0.35 | 0.66 | 0.00 | 0.01 |
|  | Left Smile Motor | 0.22 | 0.45 | 0.01 | 0.02 |
|  | Right Blink Motor | 0.26 | 0.43 | 0.01 | 0.01 |
|  | Right Grin Motor | 0.23 | 0.38 | 0.00 | 0.01 |
|  | Right Smile Motor | 0.32 | 0.58 | 0.01 | 0.01 |
| sub041 | Left Blink Motor | 0.13 | 0.39 | 0.00 | 0.01 |
|  | Left Grin Motor | 0.35 | 0.66 | 0.00 | 0.01 |
|  | Left Smile Motor | 0.22 | 0.45 | 0.01 | 0.02 |
|  | Right Blink Motor | 0.26 | 0.43 | 0.01 | 0.01 |
|  | Right Grin Motor | 0.23 | 0.38 | 0.00 | 0.01 |
|  | Right Smile Motor | 0.32 | 0.58 | 0.01 | 0.01 |
| sub042 | Left Blink Motor | 0.53 | 0.89 | 0.01 | 0.02 |
|  | Left Grin Motor | 0.38 | 0.68 | 0.01 | 0.02 |
|  | Left Smile Motor | 0.10 | 0.22 | 0.00 | 0.01 |
|  | Right Blink Motor | 0.30 | 0.49 | 0.01 | 0.02 |
|  | Right Grin Motor | 0.07 | 0.15 | 0.00 | 0.01 |
|  | Right Smile Motor | 0.17 | 0.28 | 0.01 | 0.02 |
| sub043 | Left Blink Motor | 0.19 | 0.37 | 0.00 | 0.01 |
|  | Left Grin Motor | 0.14 | 0.25 | 0.00 | 0.01 |
|  | Left Smile Motor | 0.19 | 0.32 | 0.01 | 0.01 |
|  | Right Blink Motor | 0.13 | 0.20 | 0.00 | 0.00 |
|  | Right Grin Motor | 0.17 | 0.26 | 0.00 | 0.00 |
|  | Right Smile Motor | 0.21 | 0.36 | 0.01 | 0.02 |
| sub044 | Left Blink Motor | 0.67 | 1.15 | 0.01 | 0.01 |
|  | Left Grin Motor | 0.33 | 0.59 | 0.01 | 0.02 |
|  | Left Smile Motor | 0.25 | 0.42 | 0.01 | 0.01 |
|  | Right Blink Motor | 0.62 | 1.05 | 0.01 | 0.02 |
|  | Right Grin Motor | 0.17 | 0.36 | 0.01 | 0.01 |
|  | Right Smile Motor | 0.11 | 0.21 | 0.00 | 0.01 |
| sub045 | Left Blink Motor | 0.31 | 0.47 | 0.01 | 0.01 |
|  | Left Grin Motor | 0.24 | 0.50 | 0.03 | 0.04 |
|  | Left Smile Motor | 0.40 | 0.88 | 0.02 | 0.04 |
|  | Right Blink Motor | 0.11 | 0.18 | 0.00 | 0.01 |
|  | Right Grin Motor | 0.37 | 0.66 | 0.02 | 0.03 |
|  | Right Smile Motor | 0.17 | 0.34 | 0.00 | 0.01 |
| sub046 | Left Blink Motor | 0.21 | 0.75 | 0.01 | 0.01 |
|  | Left Grin Motor | 0.34 | 0.67 | 0.01 | 0.02 |
|  | Left Smile Motor | 0.51 | 1.12 | 0.01 | 0.02 |
|  | Right Blink Motor | 0.37 | 0.61 | 0.01 | 0.02 |
|  | Right Grin Motor | 0.41 | 0.87 | 0.01 | 0.02 |
|  | Right Smile Motor | 0.62 | 1.12 | 0.01 | 0.03 |
| sub047 | Left Blink Motor | 0.11 | 0.30 | 0.02 | 0.02 |
|  | Left Grin Motor | 0.35 | 0.60 | 0.02 | 0.04 |
|  | Left Smile Motor | 0.23 | 0.40 | 0.01 | 0.01 |
|  | Right Blink Motor | 0.45 | 0.85 | 0.01 | 0.02 |
|  | Right Grin Motor | 0.53 | 1.34 | 0.05 | 0.08 |
|  | Right Smile Motor | 0.31 | 0.63 | 0.01 | 0.02 |
| sub048 | Left Blink Motor | 0.18 | 0.28 | 0.01 | 0.01 |
|  | Left Grin Motor | 0.12 | 0.25 | 0.01 | 0.01 |
|  | Left Smile Motor | 0.08 | 0.17 | 0.00 | 0.01 |
|  | Right Blink Motor | 0.43 | 0.69 | 0.02 | 0.03 |
|  | Right Grin Motor | 0.07 | 0.11 | 0.00 | 0.01 |
|  | Right Smile Motor | 0.12 | 0.23 | 0.01 | 0.01 |
| sub049 | Left Blink Motor | 0.19 | 0.37 | 0.00 | 0.01 |
|  | Left Grin Motor | 0.33 | 0.66 | 0.01 | 0.02 |
|  | Left Smile Motor | 0.34 | 0.53 | 0.01 | 0.01 |
|  | Right Blink Motor | 0.40 | 0.95 | 0.01 | 0.02 |
|  | Right Grin Motor | 0.64 | 1.50 | 0.03 | 0.05 |
|  | Right Smile Motor | 0.45 | 0.90 | 0.01 | 0.01 |
| sub050 | Left Blink Motor | 0.16 | 0.28 | 0.01 | 0.03 |
|  | Left Grin Motor | 0.88 | 1.39 | 0.02 | 0.04 |
|  | Left Smile Motor | 0.24 | 0.43 | 0.01 | 0.02 |
|  | Right Blink Motor | 0.29 | 0.53 | 0.01 | 0.02 |
|  | Right Grin Motor | 0.46 | 1.08 | 0.02 | 0.03 |
|  | Right Smile Motor | 0.37 | 0.59 | 0.01 | 0.02 |

Sup Table 4. Participant flow before and after exclusions.

| Task | ALL (before exclusions) | NC (after exclusions) | FS-LDS (after exclusions) | FS-HDS (after exclusions) |
| --- | --- | --- | --- | --- |
| Left Blink Motor | 49 | 19 | 15 | 14 |
| Left Grin Motor | 49 | 19 | 15 | 14 |
| Left Smile Motor | 49 | 19 | 16 | 14 |
| Right Blink Motor | 49 | 19 | 16 | 13 |
| Right Grin Motor | 49 | 19 | 15 | 13 |
| Right Smile Motor | 49 | 19 | 16 | 14 |
